# Research status of triglyceride glucose-body mass index (TyG-BMI index)

**DOI:** 10.3389/fcvm.2025.1597112

**Published:** 2025-07-18

**Authors:** Ke Song, Yuwan Xu, Shuairan Wu, Xiaojie Zhang, Yi Wang, Sancong Pan

**Affiliations:** ^1^ Department of Cardiovascular Medicine, Jincheng People's Hospital, Jincheng, Shanxi, China; ^2^ The First Clinical Hospital of Changzhi Medical College, Changzhi, Shanxi, China

**Keywords:** triglyceride glucose-body mass index, insulin resistance, research status, cardiovascular disease, diabetes

## Abstract

Insulin resistance (IR) represents a pivotal metabolic risk factor, with metabolic abnormalities intricately linked to increased predisposition to cardiovascular, digestive, and immune system disorders. While the triglyceride glucose (TyG) index is widely recognized as a simple and specific surrogate marker for IR, the triglyceride glucose body mass index (TyG-BMI), incorporating obesity metrics, has emerged as a more robust predictor of IR. Growing evidence underscores the strong association between TyG-BMI index and multisystem diseases that span cardiovascular, metabolic, and neoplastic pathways. Monitoring TyG-BMI index enables proactive management of lifestyle modifications, dietary interventions, and physical health strategies, thereby reducing disease prevalence. This review synthesizes the pathophysiological mechanisms underlying TyG-BMI, alongside its clinical utility and cutting-edge research advancements in hypertension, coronary artery disease, stroke, diabetes, non-alcoholic fatty liver disease, hyperuricemia, and cancer. Particular emphasis is placed on the role of TyG-BMI index in influencing disease progression, highlighting its potential as a transformative biomarker for risk stratification and therapeutic targeting across diverse medical disciplines.

## 1 Introduction

In modern society, with the increase in stress levels and the improvement in material living standards, cardiovascular diseases, metabolic disorders and cancer have emerged as the most significant challenges and burdens for global healthcare systems (1). Obesity, a core component of metabolic syndrome, not only predisposes individuals to cardiovascular disease but also serves as a critical risk factor. Body Mass Index (BMI), a widely used metric in epidemiological surveys and population health management, serves as a standard tool for assessing obesity (2). Notably, plasma triglyceride (TG) levels, blood glucose concentrations, and BMI are intricately interconnected through the interplay of adipose tissue, muscle function, and pancreatic β-cell activity (3). When these three parameters are disrupted concurrently, due to their interdependence in regulating energy homeostasis, the body's susceptibility to disease increases significantly, underscoring the importance of holistic metabolic assessment in preventive medicine.

**Table 1:** The correlation between TyG-BMI index and various diseases.

Disease category	Title	Author/Year	Research design	Result
HTN	Association of triglyceride glucose index and its combination of obesity indices with prehypertension in lean individuals: A cross-sectional study of Chinese adults.	Zeng et al. (24)	Cross-sectional study	A analysis was conducted on 105,070 non-hypertensive lean adults. It was found that the mean values of triglyceride-glucose (TyG) index, TyG-BMI index, TyG-waist circumference (TyG-WC), and TyG-waist-to-height ratio (TyG-WHtR) in the prehypertensive population were higher than those in normotensive individuals. All four indicators were positively correlated with systolic blood pressure and diastolic blood pressure. After full adjustment, only TyG-BMI index and TyG-WC were significantly associated with prehypertension in both men and women, with TyG-BMI index showing the highest odds ratio (OR) for prehypertension. The study suggests that TyG-BMI index may serve as a convenient supplementary monitoring indicator for the stratified management of prehypertensive patients without obesity.
HTN	Predicting hypertension by obesity- and lipid-related indices in mid-aged and elderly Chinese: a nationwide cohort study from the China Health and Retirement Longitudinal Study.	Li et al. (25)	Cohort study	In a four-year study among middle-aged and elderly Chinese populations, the incidence of hypertension was 22.08% in men and 17.82% in women. Thirteen obesity- and lipid-related indices were analyzed, among which the TyG-BMI index showed predictive value for hypertension: In men: odds ratio (OR) = 2.085, 95% confidence interval (CI) = 1.671–2.602, *P* < 0.05. In women: OR = 2.264, 95% CI = 1.803–2.843, *P* < 0.05.
HTN	Correlation between triglyceride glucose-body mass index and hypertension risk: evidence from a cross-sectional study with 60,283 adults in eastern China.	Chen et al. (26)	Cross-sectional study	TyG-BMI index, an effective indicator for assessing IR, is positively correlated with hypertension. The TyG-BMI index in the hypertension group is significantly higher than that in the healthy group. TyG-BMI index (OR: 1.61/SD increased; 95% CI: 1.55–1.67, *P* < 0.05); ROC (TyG-BMI index cut-off value: 207.105, AUC: 0.719, sensitivity 65.5%, specificity 66.8%)
HTN	Association between different insulin resistance surrogates and all-cause mortality in patients with coronary heart disease and hypertension: NHANES longitudinal cohort study.	Hou et al. (27)	Longitudinal cohort study	This study did not find a significant correlation between TyG-BMI index and the prognosis of HTN patients with coronary heart disease, but there was a significant difference in TyG-BMI index between the survival group and the death group (*P* < 0.001).
CHD	Comparison of Innovative and Traditional Cardiometabolic Indices in Estimating Atherosclerotic Cardiovascular Disease Risk in Adults	Huang et al. (29)	Cross-sectional study	The study included 3,143 Taiwanese adults aged 20–79 years, with elevated risk defined as a 10-year ASCVD risk ≥7.5% using the Pooled Cohort Equations. Multivariable-adjusted logistic regression analysis showed that all cardiometabolic indices (*P* < 0.001) were significantly associated with elevated ASCVD risk, except for the body shape index (ABSI) and conicity index (CI) in women. The association between TyG-BMI index and 10-year ASCVD risk.
CHD	Selenium Concentration Is Positively Associated with Triglyceride-Glucose Index and Triglyceride Glucose-Body Mass Index in Adults: Data from NHANES 2011–2018.	Li et al. (30)	Cross-sectional study	Serum selenium level is positively correlated with TyG-BMI index, and excessive blood selenium may be related to an increased risk of cardiovascular disease. TyG-BMI index (*β*: 3.185, 95% CI: 2.102–4.268, *P* < 0.05).
CHD	Association between triglyceride glucose-body mass index and cardiovascular outcomes in patients undergoing percutaneous coronary intervention: a retrospective study.	Cheng et al. (32)	Retrospective study	This study showed that a higher TyG-BMI index was proportionally associated with an increased incidence of MACCE in elderly or female patients. Multivariate logistic regression analysis showed that there was a linear relationship between TyG-BMI index and MACCE: elderly patients (OR: 1.22, 95% CI: 1.011–1.467, *P* = 0.038);female patients (OR = 1.33, 95% CI: 1.004–1.764, *P* = 0.047). However, the inclusion of TyG-BMI index does not better predict MACCE in elderly patients (especially female patients).
CHD	Association between the cumulative average triglyceride glucose-body mass index and cardiovascular disease incidence among the middle-aged and older population: a prospective nationwide cohort study in China.	Li et al. (33)	Prospective nationwide cohort study in China	The study data were derived from the CHARLS, with a final inclusion of 7,376 participants. The research found a positive linear association between elevations in the t TyG-BMI index and the incidence of CVD. Specifically, each 10-unit increase in TyG-BMI index was significantly associated with an elevated risk of CVD. During the follow-up period, 833 participants were diagnosed with CVD, and higher quartiles of TyG-BMI index were associated with higher risk predictions. Additionally, the study revealed significant differences only across age groups, suggesting that TyG-BMI index may serve as an important reference for assessing cardiovascular disease risk in patients with CKM syndrome.
AF	The association between triglyceride glucose-body mass index and all-cause mortality in critically ill patients with atrial fibrillation: a retrospective study from MIMIC-IV database.	Hu et al. (39)	Retrospective study	Lower TyG-BMI index levels were significantly associated with a higher risk of all-cause mortality at 30 days, 90 days, 180 days, and 365 days in critically ill patients with atrial fibrillation. The TyG-BMI index showed an L-shaped relationship with all-cause mortality, with inflection points of 223.60 for 30-day all-cause mortality and 255.02 for 365-day all-cause mortality. Compared with patients with TyG-BMI index levels below the inflection points, those with higher TyG-BMI index levels had a 1.8% lower risk of 30-day all-cause mortality and a 1.1% lower risk of 365-day all-cause mortality. The TyG-BMI index can serve as an effective indicator for the stratification and treatment of atrial fibrillation patients in the ICU.
HF	Association between triglyceride glucose-body mass and one-year all-cause mortality of patients with heart failure: a retrospective study utilizing the MIMIC-IV database.	Dou et al. (41)	Retrospective study	HF patients with higher TyG-BMI index had a significantly lower risk of death, showing an “L"-shaped association with survival. TyG-BMI index had a moderate predictive ability for 1-year mortality, with an AUC of 0.647 (0.581, 0.715). The optimal cutoff value was 244.35, with a specificity of 59.2% and a sensitivity of 67.1%. Patients in the lowest quartile of TyG-BMI index had the highest risk of 1-year all-cause mortality. Compared with the lowest TyG-BMI index group, the hazard ratio from the fully adjusted Cox model was 0.24 (95% CI: 0.10, 0.59; *P* = 0.002).
HF	Association between triglyceride glucose-body mass index and long-term adverse outcomes of heart failure patients with coronary heart disease.	Lyu et al. (42)	Prospective cohort study	The TyG-BMI index can be used as an important prognostic marker for all-cause mortality and HF readmission rate in HF patients with CHD. Threshold analysis showed that there was an inverted “J” relationship between TyG-BMI index and all-cause mortality, and the cut-off point was 240.0 (correction model: HR: 0.90, 95% CI:0.86–0.93; *P* = 0.003). The relationship between and HF readmission was a “U”-shaped relationship, with an inflection point of 228.56 (adjusted model: the following figure: HR: 0.95, 95% CI: 0.91–0.98; the figure above: HR: 1.08, 95% CI: 1.03–1.13; *P* < 0.001).
Ischemic	Estimate of prevalent ischemic stroke from triglyceride glucose-body mass index in the general population.	Du et al. (45)	Cross-sectional study	There was a strong correlation between TyG-BMI index and ischemic stroke without a saturation effect, with a 20% increase in the risk of ischemic stroke for each standard deviation (SD) increase in TyG-BMI index (OR: 1.20, 95% CI: 1.10–1.32, *P* < 0.05).
Ischemic	Changes in the triglyceride glucose-body mass index estimate the risk of stroke in middle-aged and older Chinese adults: a nationwide prospective cohort study.	Huo et al. (47)	Nationwide prospective cohort study	TyG-BMI index was independently associated with the risk of stroke in middle-aged and elderly people. Compared with the L-TyG-BMI index group, M- TyG-BMI index group (OR: 1.01; 95% CI: 0.65–1.57), H-TyG-BMI index group (OR: 1.62; 95% CI: 1.11–2.32).
Ischemic	Correlation of TyG-BMI and TyG-WC with severity and short-term outcome in new-onset acute ischemic stroke.	Yu et al. (48)	Cross-sectional study	TyG-BMI index is associated with the severity and short-term outcome of new acute ischemic stroke. With the increase of TyG-BMI index, the short-term prognosis of stroke patients will be better.
Diabetes	Triglyceride glucose—body mass index in identifying high—risk groups of pre—diabetes.	Jiang et al. (51)	Large longitudinal cohort study	There is an independent positive correlation between TyG-BMI index and prediabetes, especially among people under 50 years old, non-obese people, and women.
Diabetes	Triglyceride glucose-body mass index and the risk of diabetes: a general population-based cohort study.	Wang et al. (52)	Cohort study	TyG-BMI index is associated with diabetes, especially in young and middle-aged people and non-obese people. The TyG-BMI index was an independent predictor of new-onset diabetes (HR: 1.50, 95% CI: 1.40–1.60, *P* < 0.01), and the optimal cut-off value was 213.2966(area under the curve 0.7741, sensitivity 72.51%, specificity 69.54%).
Diabetes	Triglyceride Glucose-Body Mass Index and Risk of Incident Type 2 Diabetes Mellitus in Japanese People With Normal Glycemic Level: A Population-Based Longitudinal Cohort Study.	Song et al. (53)	Longitudinal Cohort Study	There was a positive correlation between TyG-BMI and the risk of T2 DM events, especially in young people, women, non-hypertensive people and non-drinkers. The optimal threshold of TyG-BMI index for predicting T2DM was 197.2987, with a sensitivity of 74.16% and a specificity of 68.36%.
Diabetes	Predictive Effect of Triglyceride Glucose−Related Parameters, Obesity Indices, and Lipid Ratios for Diabetes in a Chinese Population: A Prospective Cohort Study.	Li et al. (54)	Prospective Cohort Study	TyG-BMI index is an effective marker for predicting diabetes in IFG group (HR: 2.53; 95% CI: 1.97–3.26; *P* < 0.05).
Diabetes	The impact of triglyceride glucose-body mass index on all-cause and cardiovascular mortality in elderly patients with diabetes mellitus: evidence from NHANES 2007–2016.	Ding et al. (55)	Cross-sectional study	There was a U-shaped correlation between TyG-BMI index and all-cause mortality in elderly patients, and a linear correlation between TyG-BMI index and cardiovascular mortality.
HUA	Associations of Triglyceride-Glucose Index and Its Derivatives with Hyperuricemia Risk: A Cohort Study in Chinese General Population.	Gu et al. (57)	Cohort study	TyG-BMI contributes to risk stratification and prevention of HUA, and the TyG-BMI index was more harmful for HUA in women than in men in the diseased group compared to the normal group.Women:TyG-BMI index: HR: 2.053, 95% CI: 1.520–2.773, *P* < 0.001; Men: TyG-BMI index: HR: 1.271, 95% CI: 1.099–1.469, *P* < 0.001.
HUA	Insulin resistance surrogates predict hypertension plus hyperuricemia.	Li et al. (59)	Cross-sectional epidemiological study	TyG-BMI index was associated with hypertension with hyperuricemia, AUC: 0.72, 95% CI: 0.70–0.74.
NAFLD	Association between triglyceride glucose-body mass index and non-alcoholic fatty liver disease in the non-obese Chinese population with normal blood lipid levels: a secondary analysis based on a prospective cohort study.	Li et al. (63)	Cohort study in observational research	TyG-BMI may have clinical significance in identifying groups at high risk of NAFLD. TyG-BMI index HR: 3.09, 95% CI: 2.63–3.63. The predictive value of TyG-BMI for the incidence of NAFLD ROC: 0.85, 95% CI: 0.84–0.86, specificity 0.73, sensitivity 0.82.
NAFLD	Triglyceride glucose-body mass index is effective in identifying nonalcoholic fatty liver disease in nonobese subjects.	Zhang et al. (65)	Cross-sectional study	TyG-BMI index is an effective index to detect NAFLD in non-obese people. TyG-BMI index: OR: 3.4, 95% CI: 3.0–3.9.
NAFLD	Usefulness of the triglyceride glucose-body mass index in evaluating nonalcoholic fatty liver disease: insights from a general population.	Wang et al. (66)	Cross-sectional study	The TyG-BMI index was independently and positively correlated with NAFLD. TyG-BMI index: OR: 3.90, 95% CI: 3.54–4.29,*P* < 0.01.
NAFLD	The triglyceride glucose-body mass index: a non-invasive index that identifies non-alcoholic fatty liver disease in the general Japanese population.	Hu et al. (67)	Cross-sectional study	The TyG-BMI index has been shown to be a diagnostic tool for assessing NAFLD. When the TyG-BMI index is 182.2, NAFLD can be excluded with high accuracy, and the TyG-BMI index is 224.0, which can effectively determine the presence of NAFLD.
NAFLD	Association between triglyceride-glucose related indices and mortality among individuals with non-alcoholic fatty liver disease or metabolic dysfunction-associated steatotic liver disease.	Chen et al. (69)	Prospective cohort study	The study analyzed 10,390 participants based on data from the National Health and NHANES and the NDI databases, among whom 3,672 were diagnosed with NAFLD and 3,556 with MASLD. The results showed that both TyG-BMI index and TyG-WC indices were significantly positively associated with all-cause mortality, cardiovascular mortality, and diabetes-related mortality. The associations between TyG-BMI index/TyG-WC indices and all-cause mortality were more pronounced in patients without progressive hepatic fibrosis.
Metabolic bone disease	Triglyceride Glucose–Body Mass Index Is a Reliable Indicator of Bone Mineral Density and Risk of Osteoporotic Fracture in Middle-Aged and Elderly Nondiabetic Chinese Individuals.	Wen et al. (70)	Prospective study	The TyG-BMI index was negatively correlated with fracture risk in Chinese non-diabetic middle-aged and elderly men and women.
Metabolic bone disease	A negative association between triglyceride glucose-body mass index and testosterone in adult males: a cross-sectional study.	Wu et al. (74)	Cross-sectional study	There is a negative correlation between TyG-BMI index and testosterone in adult males. The increase of TyG-BMI index and the decrease of testosterone level may indirectly lead to the decrease of bone mineral density and the occurrence of osteoporosis.
Cancer	Association of triglyceride glucose-body mass index with non-small cell lung cancer risk: A case-control study on Chinese adults.	Wang et al. (76)	Case-control study	TyG-BMI index is a useful tool for assessing NSCLC risk. OR: 1.014; 95% CI: 1.007–1.021; *P* < 0.001.
Cancer	Increased pretreatment triglyceride glucose-body mass index associated with poor prognosis in patients with advanced non-small cell lung cancer.	Guo et al. (77)	Retrospective study	TyG-BMI and poor prognosis in patients with advanced NSCLC are more important in smoking and high CRP.
Cancer	Association between four insulin resistance surrogates and the risk of esophageal cancer: a prospective cohort study using the UK Biobank.	Yang et al. (78)	Prospective cohort study	TyG-BMI index is closely related to the risk of EAC and ESCC. For each standard deviation increase in the TyG-BMI index, the risk of EAC increased by 1.37, while the risk of ESCC decreased by 0.67.
Cancer	Prognostic significance of glucose-lipid metabolic index in pancreatic cancer patients with diabetes mellitus.	Wang et al. (79)	Retrospective analysis	TyG-BMI index has a prognostic effect on PC with diabetic. TyG-BMI index: HR: 1.92; 95% CI: 1.33–2.76; *P* < 0.01).

TyG-BMI, Triglyceride glucose body mass index; ROC, Receiver operating characteristic; 95% CI, 95%confidence interval; AUC, Area Under Curve; CHARLS, China Health and Retirement Longitudinal Study. NHANES, National Health and Nutrition Examination Survey; NDI, National Death Index; RCS, Restricted cubic splines; CHD, Coronary heart disease; PCI, Percutaneous coronary intervention; DES, Drug-eluting stent; MACCE, Major adverse cardiac and cerebrovascular events; CHARLS, China Health and Retirement Longitudinal Study. MIMIC-IV, Medical Information Mart for Intensive Care IV; NCRCHS, Northeast China Rural Cardiovascular Health Study; NSSIPL, National Stroke Screening and Intervention Program in Liaoning; IFG, Impaired fasting glucose; NHANES, National Health and Nutrition Examination Survey; HUA, hyperuricemia; NAFLD, Nonalcoholic fatty liver disease; WISCO, Wuhan Iron and Steel Company; NSCLC, Non-small-cell lung cancer; CRP, C reactive protein; EAC, esophageal adenocarcinoma; ESCC, esophageal squamous cell carcinoma; UKB, United Kingdom Biobank; PC, Pancreatic cancer. ASCVD, atherosclerotic cardiovascular disease; CKM, cardio-renal-metabolic; AS, atherosclerosis, AF, atrial fibrillation; HF, heart failure; HT, hypertension; MI, myocardial infarction; T2DM, Type 2 diabetes; HUA, hyperuricemia; NAFLD, Non-alcoholic fatty liver disease.

Insulin resistance (IR) is intimately linked to disorders across multiple organ systems, including cardiovascular, neurological, urological, endocrine, gastrointestinal, and neoplastic conditions. The pathophysiological correlation between IR and these multisystem disorders is profound. IR is a central metabolic abnormality characterized by reduced sensitivity to insulin (both endogenous and exogenous), which is associated with endoplasmic reticulum (ER) stress, oxidative stress, activation of proinflammatory cytokines, dysregulated glucose, and lipid metabolism (3). This leads to impaired glucose tolerance, reduced glucose uptake by cardiomyocytes, diminished myocardial inotropy, disruption of intracellular signaling pathways, and compromised neural function (4). On the other hand, this promotes cell proliferation, inhibits apoptosis, and triggers inflammation, ultimately leading to cancer development (5). The hyperinsulinemic-euglycemic clamp (HEGC), the gold standard for measuring IR, is difficult to apply in large population studies and clinical settings due to its high cost, time-consuming nature, and complexity. In response to this limitation, the homeostatic model of insulin resistance (HOMA-IR) has been proposed to assess β-cell function and IR, but it requires fasting insulin measurements and is less suitable for general population screening (6–8). Given these limitations, using IR-sensitive biomarkers as clinical tools for evaluating metabolic health may offer practical advantages in research and clinical practice.

Currently utilized to evaluate IR, the triglyceride glucose index (TyG) is a crucial indicator of HEGC and HOMA-IR derived from TG and fasting plasma glucose (FPG) levels (9). According to recent research, a measure of IR may also be the TyG-BMI index, which combines the anthropometric BMI and the TyG index. We shall methodically outline the TyG-BMI index's development history in this review. We will also go over recently released research that has clarified the essence of the TyG-BMI index in a number of systemic disorders as well as the underlying mechanisms behind it.

## 2 Methods

**Figure 1:**
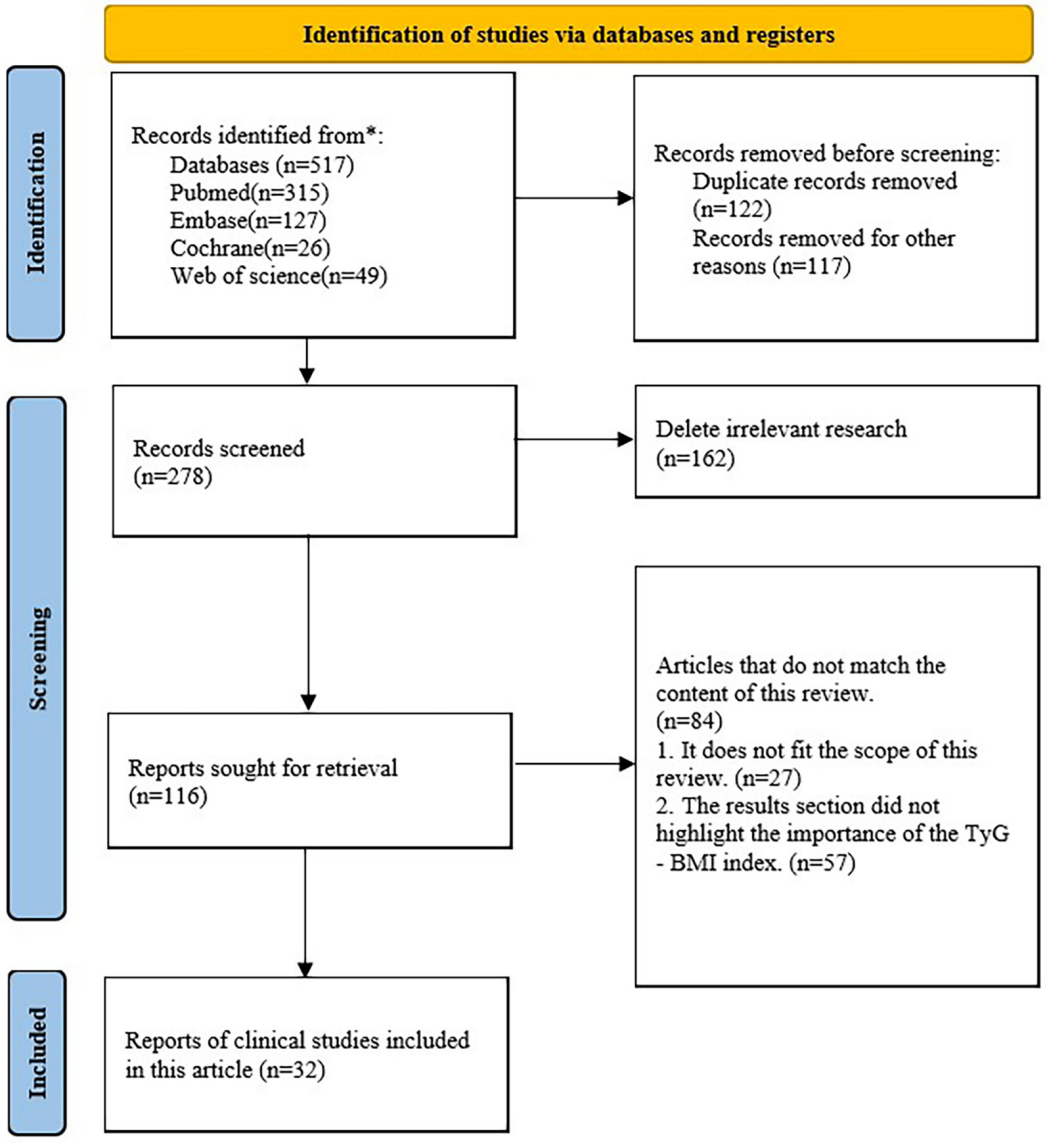
Flowchart for database retrieval and research identification.

The TyG-BMI index is rigorously assessed in terms of a range of gastrointestinal, tumor-related, neurologic, urologic, circulatory, and endocrine disorders. Both observational and retrospective studies including clinical populations with various clinical characteristics were included in the selection of studies. Eligible studies were not limited by time or language. The electronic databases Web of Science, PubMed, Embase, and Cochrane were used for screening. Triglyceride-glucose-body mass index, or TyG-BMI index, and coronary heart disease, hypertension, heart failure, atrial fibrillation, stroke, hyperuricemia, osteoporosis, tumor, diabetes, or fatty liver disease are some of the search terms that may be used. The authors evaluated these studies' suitability after retrieving their complete texts (Figure 1).

## 3 Triglyceride glucose-body mass index

TyG-BMI index is a reliable, sensitive and specific surrogate marker of IR, which is associated with fasting plasma glucose (FPG), serum triglyceride (TG) and body mass index (BMI), calculated as Ln [TG (mg/dl) × FPG (mg/dl)/2] × BMI. This index was first proposed in 2016 by Er et al. (10) by comparing various lipid parameters: lipid levels and ratios, visceral adiposity index (VAI) and Lipid accumulation products (LAP), adipokine levels and ratios, TyG index, TyG-BMI index, Waist Circumference (WC), Waist-to-Height Ratio (WTHR) for identification of IR; a conclusion was made that the TyG-BMI index is an alternative marker that can be used as an alternative marker of IR, which is simple useful and simple to apply in clinical settings; it was also noted that, with a range of 16.6%, the TyG-BMI index had a strong correlation with HOMA-IR among the visceral obesity indices and TyG-related values. According to a study by Mir et al. (11) that compared eight IR-related indexes in patients without diabetes, all of the indexes were diagnostically performed. The indexes were grouped according to BMI: normal (BMI: 18.5–24.9 kg/m^2^), overweight (BMI: 24.9–29.9 kg/m^2^), and obese (BMI ≥ 30 kg/m^2^). The normal group's diagnostic IR performance was highest for the TyG index (0.909) and TyG BMI index (0.879). A Study by Lim et al. (12) not only confirmed the above findings, but further suggested that TyG-BMI index is a more accurate predictor of IR than TyG index and TyG-WC index alone. It can be seen that BMI in combination with TyG index can comprehensively identify and measure obesity and metabolic abnormalities in an individual (13), which is composed of three classical metabolic indices related to lipids, blood glucose, and obesity, and it is a reliable indicator for predicting.

**Figure 2:**
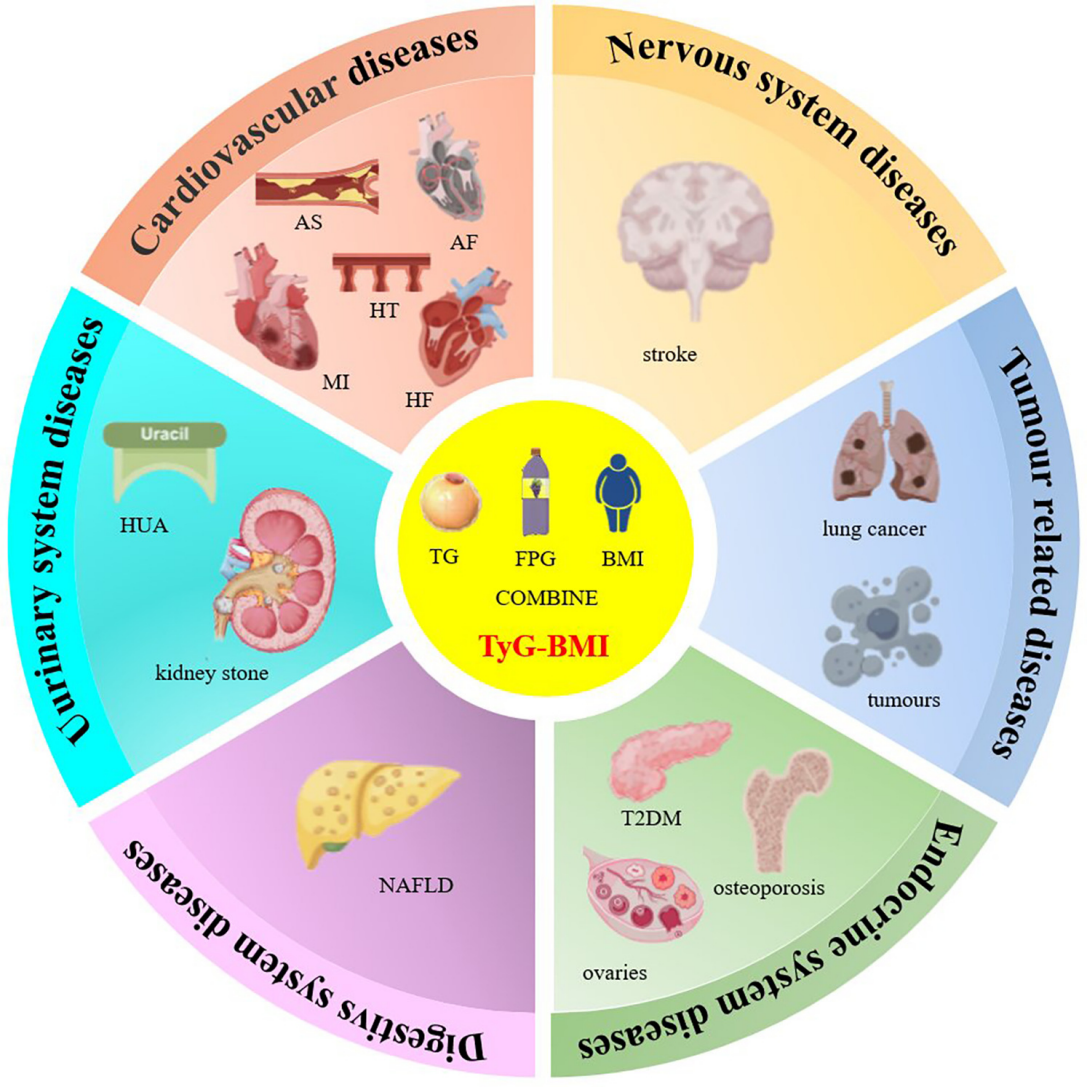
TyG-BMI index and multisystem diseases. Note: AS: atherosclerosis, AF: atrial fibrillation; HF: heart failure; HT: hypertension; MI: myocardial infarction; T2DM: Type 2 diabetes; HUA: hyperuricemia; NAFLD: Non-alcoholic fatty liver disease.

Primarily, dyslipidemia lacks proper insulin signaling, especially in peripheral tissues such as adipocytes, leading to disorders of lipid metabolism, where higher TG levels can induce elevated plasma free fatty acid (FFA) levels, leading to increased production of reactive oxygen species (ROS) as well as inflammation and apoptosis induced by the protein kinase C (PKC) signaling pathway; secondly, glucose metabolism is disturbed, leading to hyperglycemia and ultimately triggers inflammation and oxidative stress, leading to inactivation of nitric oxide (NO) and generation of overproduced ROS, impairing endothelial function; finally, obesity presents a chronic inflammatory state with an imbalance between pro-inflammatory and anti-inflammatory immune cells, and also leads to elevated levels of monocytes, which are positively correlated with the degree of IR (14–18). All of these factors, when combined, triggers oxidative stress and endothelial dysfunction, which promotes the development of atherosclerotic cardiovascular and metabolic diseases. In addition, the overproduction of reactive oxygen species in insulin-resistant individuals increases the risk of cancer, causes DNA damage and mutagenesis, and causes carcinogenesis (19, 20). In addition, the large number of inflammatory cells in the adipose tissue of obese and diabetic patients predisposes them to tumorigenesis. In this article, we will discuss the progress and significance of TyG-BMI index research (Figure 2).

## 4 TyG-BMI index and cardiovascular disease

### 4.1 TyG-BMI index and hypertension and prehypertension

Studies have demonstrated that blood pressure ≥ 130/80 mmHg (1 mmHg = 0.1333 kPa) is associated with a 35% increase in coronary heart disease events, a 56% increase in cardiovascular deaths, a 95% increase in strokes, and a 99% increase in myocardial infarctions (21). Additionally, obesity favors is a risk factor of hypertension (HTN), which can result in IR or hyperinsulinemia, elevated leptin, enhanced renal sodium reabsorption, and improved functioning of the sympathetic nervous system and renin-angiotensin-aldosterone system activity. In IR (22), the phosphatidylinositol-3-kinase PI3K pathway results in a decrease in NO production, while the mitogen-activated protein kinase (MAPK) pathway is triggered, which causes vasoconstriction (23), causing the development of HTN. HTN has become the most important risk factor for the occurrence of cardiovascular disease. In the early stage of elevated blood pressure, spasmodic contraction of small arteries and impaired elasticity of large arteries occur. The formation and progression of atherosclerosis ultimately damages target organs resulting in conditions such as myocardial hypertrophy, coronary artery stenosis, cerebrovascular lesions and glomerulosclerosis that ultimately can have serious consequences if so advanced as cardiovascular disease, stroke and renal failure (Table 1).

Prehypertension in normal weight persons was positively correlated with obesity status, TyG-BMI index, and TyG-WC index, according to large cross-sectional research that examined and analyzed 105,070 adults with normal weight and no hypertension (24). Furthermore, the area under the curve (AUC) and the TyG-BMI index odds ratio (OR) outperformed the other parameters. Using binary logistic regression, Li et al. (25) investigated the link between 13 obesity-related anthropometric indices and hypertension. Of these, 13 indices, including the TyG-BMI index, demonstrated moderate predictive ability (AUC > 0.5) and were significant in predicting hypertension in young and older Chinese adults. Through the big data study of 60,283 subjects in eastern China, Chen et al. confirmed that TyG-BMI index is independently associated with hypertension (26), especially in young and middle-aged people. However, in a recent longitudinal cohort study (27), no significant correlation was found between TyG-BMI index and the prognosis of patients with HTN combined with coronary artery disease. Nonetheless, we continue to hold the view that there is a positive association between the two, based on several prior clinical investigations and the pathophysiology of the TyG-BMI index and HTN. In order to prevent and manage hypertension or prehypertension, the TyG-BMI index, as a surrogate marker of IR, is a straightforward and clinically useful indicator. Additionally, biochemical markers can be monitored at an early stage through dietary, lifestyle, and medication changes to control blood pressure and lower the risk of organ damage.

### 4.2 TyG-BMI index and coronary atherosclerotic heart disease (CHD)

Atherosclerosis, inflammation, and embolism of the coronary vessels lead to narrowing or blockage of the lumen, which results in myocardial ischemia, hypoxia, or necrosis collectively known as coronary atherosclerotic heart disease (CHD). The main contributing variables include diabetes, dyslipidemia, hypertension, obesity, and a sedentary lifestyle (28). IR triggers the proliferation of vascular smooth muscle cells and collagen cross-linking deposits leading to the reduction of arterial elasticity and plaque and calcification formation, which is the main cause of lipid metabolism disorder related IR and cardiovascular disease.

In their investigation, Huang et al. (29) investigated risk of developing atherosclerotic cardiovascular disease (ASCVD) in 3,143 Taiwanese volunteers between the ages of 20 and 79. They revealed that the TyG-BMI index was substantially linked to a high risk of ASCVD in both men and women. A unique study suggested (30) a positive correlation between blood selenium levels and TyG-BMI index; it is well known that selenium prevents atherosclerosis by modulating the process of inflammation, inhibiting oxidative stress, and protecting endothelial cells from apoptosis (31), but excessive selenium may interfere with insulin signaling, acting in the opposite direction; elevated blood selenium leads to an increased probability of atherosclerosis, which indirectly proves that there is an association of elevated TyG-BMI index and atherosclerosis. A study in China investigated the value of TyG index and TyG-BMI index in the prediction and assessment of CHD, and the results showed that TyG index (95% CI: 0.64–0.79, *P* < 0.05) and TyG-BMI index (95% CI: 0.61–0.76, *P* < 0. 05) were higher in patients with CHD than those with non-CHD, and both of them were independent influences on CHD development after multifactorial Logistic regression analysis; TyG index and TyG-BMI index were higher in those with moderate and severe Gensini scores than in those with mild lesions; meanwhile, SYNTAX II scores were higher in high-risk than in low-risk patients (*P* < 0.05). According to Cheng et al. (32), a higher TyG-BMI index following the implantation of drug-eluting stents (DES) was closely associated with a higher incidence of major cardiovascular diseases in older and female patients. In both young adults and older people, there was an evident and significant correlation between cumulative mean TyG-BMI index and CVD events, according to data analysis based on the China Health and Aging National Tracking Survey (CHARLS) (33). Therefore, TyG-BMI index may provide new ideas for risk stratification and CHD. However, clinical studies in this area are still relatively few, and the correlation between TyG-BMI index and coronary heart disease needs to be further investigated and validated by large-sample, multicenter studies.

### 4.3 TyG-BMI index and atrial fibrillation

Atrial fibrillation (AF) is a common arrhythmia disease where a rapid and disorganized rhythm replaces a normal rhythm. Currently, there are more studies on the association of IR with diseases in terms of atherosclerosis, while the correlation with AF is still controversial. As mentioned above, there is a strong correlation between TyG-BMI index and IR. Previous studies have shown that IR-induced systemic inflammatory response and oxidative stress can induce atrial remodeling, inflammatory spread, local myocardial fibrosis, and calcium homeostasis impairment, which can lead to cardiac conduction abnormalities and atrial fibrillation (34). And transforming growth factor-β1 (TGF-β1) is also an important mediator of atrial remodeling (35). IR directly affects the expression of TGF-β1 in rat cardiomyocytes and fibroblasts, which promotes myocardial interstitial fibrosis and leads to AF (36).

In an early 10-year community follow-up study, no correlation was revealed in the case of IR and AF events as the average age of the studied population was low (59 years old). Also, the prevalence of cardiovascular risk factors was reduced (37). However, Chan et al. observed the correlation between IR and AF after 15 weeks of feeding three groups of mice (normal diet group, high fat group, high cholesterol and fructose group), and concluded that the promoting factors of IR (high fat, high sugar and high cholesterol) can lead to atrial interstitial fibrosis and abnormal calcium homeostasis, change the conduction velocity of myocardial cells and increase the ectopic activity of atrium, which is helpful to the occurrence of AF (36). In Maria et al., it was also concluded that IR caused impaired glucose transport in the atrium by feeding two groups of male mice (fed on normal diet group and high fat diet group), which may provide metabolic pro-inflammatory substrates and become a new early pathogenic factor of AF (38). Different from the study of Chan et al., it is shown that AF can still occur when there is no fibrosis in the atrium (36). Hu et al. first proved that TyG-BMI index is an effective index for the classification and treatment of severe AF patients by calculating the level of TyG-BMI index (39). The above studies have shown that there is a positive correlation between cumulative metabolic burden and the risk of atrial fibrillation. It can be seen that TyG-BMI index, an alternative marker of IR, is expected to become an effective tool for AF prediction. For AF patients, it is still necessary to strictly control the metabolic indicators to maintain normal or lower levels, which helps to avoid the occurrence and development of the disease.

### 4.4 TyG-BMI index and heart failure

Heart failure (HF) is the terminal stage in many cardiac diseases, which seriously affects the quality of patients' survival and its mortality rate is extremely high. With the increase in heart diseases such as HTN, AF and CAD, the number of people suffering from HF is increasing and younger. HF is not all caused by organic heart disease, irregular life and diet, alcoholism, high fat, high glucose, and obesity can impact the normal function of the heart. When the TyG-BMI index increases, individuals with IR are more prone to HF. IR leads to the excessive activation of the renin-angiotensin-aldosterone system (RAAS). Aldosterone and angiotensin II (AngII) stimulate the NADPH oxidase complex, subsequently activating vascular smooth muscle, cardiac, and skeletal muscle tissues. This process triggers the production of a large amount of ROS, activates protein kinase C, S6 kinase, and mitogen-activated protein kinase (MAPK), and inhibits the insulin receptor (IRS-1), thus suppressing the normal transmission of the insulin signaling pathway (40). These two form a vicious cycle that worsens HF.

In the recent years, there has been little correlation between HF and TyG—BMI index proposed, with relatively few research articles. Exploring the relationship between TyG-BMI index and their 360-day risk of death in HF patients, Dou et al. proposed that higher levels of TyG-BMI index are associated with lower mortality (41). A single-centre prospective cohort study with a follow-up of 9.4 years noted the association of TyG-BMI index with adverse outcomes in HF with CHD (42), demonstrating a reduced risk of all-cause mortality with a TyG-BMI index of less than 240.0, and a significant inverse “J”-shaped relationship between baseline TyG-BMI index and all-cause mortality, and a “U”-shaped relationship with readmission for HF. The explanations for the negative association of TyG-BMI index with all-cause mortality were shown in several of the above studies. Considering the possible association with high BMI, higher BMI is strongly associated with a reduced risk of mortality (43). Since patients with HF are usually in a state of depletion, obesity or overweight may result in the presence of a more adequate physiological reserve, thus favouring the recovery of the organism. This phenomenon is known as the “obesity paradox” (44). Regardless, an increase in TyG-BMI index, which causes damage to both the heart and cardiomyocytes, can be a favorable indicator for assessing the prognosis of patients with HF.

## 5 TyG-BMI index and stroke

Stroke is a major challenge to healthcare systems worldwide and a leading cause of death and disability in many countries. Ischemic strokes the most common type of pathology and may be triggered by cerebral vasospasm, platelet aggregation, endothelial stripping, as well as hypertension, diabetes, and hyperlipidemia (45). Ischemic stroke and coronary heart disease are like “two melons on the same vine”. Both are caused by endothelial dysfunction, which promotes the migration and proliferation of vascular smooth muscle cells. They share the pathological basis of atherosclerosis and risk factors (46).

By including 10,862 subjects from the National Study of Cardiovascular Health in Northeastern China (NSSIPL) and 11,097 subjects from the National Stroke Screening and Intervention Program in Liaoning Province (NCRCHS), Du et al. (30) found that the level of TyG-BMI index was significantly higher, the prevalence of overweight/obesity, diabetes mellitus, dyslipidemia, coronary heart disease, and ischemic stroke was significantly higher in subjects with higher TyG-BMI index levels (*p* < 0.05). A linear correlation between TyG-BMI index levels and ischemic stroke was confirmed in both investigations and did not have a saturation effect, with a 20% increase in the risk of ischemic stroke for each standard deviation (SD) increase in TyG-BMI index. The ability of adding TyG-BMI index to predict ischemic stroke and improve risk stratification for ischemic stroke was significantly higher compared with conventional risk factors. A prospective cohort study proposed that changes in TyG-BMI index could assess stroke risk in a middle-aged and elderly Chinese population [mean age 58.68 (9.51) years] (47). Yu et al. (48) identified that TyG-BMI index was associated with severity and short-term outcome of new acute ischemic stroke. The initiating factor for ischemic stroke is atherosclerosis, which confirms that controlling all metabolic indicators in the appropriate range significantly reduces the risk stratification of ischemic stroke as TyG-BMI index decreases.

## 6 TyG-BMI index and diabetes

Diabetes mellitus is a condition characterized by impaired blood glucose metabolism, mainly due to insufficient insulin secretion or IR leading to elevated blood glucose. IR is present in patients with prediabetes, and as it gets worse, it eventually develops into T2DM. IR refers to a process in which mutations in insulin receptor genes lead to decreased receptor affinity, reduced receptor number, or intracellular signaling abnormalities (such as impairment of the classical PI3K/Akt signaling pathway), ultimately decreasing insulin sensitivity (49). Additionally, in patients with T2DM, inflammatory responses and oxidative stress promote the formation and progression of IR while interfering with insulin signaling. Studies have found that multiple pro-inflammatory cytokines–primarily tumor necrosis factor-α (TNF-α), interleukin-1β (IL-1β), and interleukin-6 (IL-6)—establish complex links between inflammation and insulin resistance by activating insulin signaling pathways such as suppressor of cytokine signaling (SOCS), PKC, and extracellular regulated protein kinase (ERK) (16, 50). This interference disrupts normal insulin transduction, reduces glucose utilization, and exacerbates IR. Initially, the body can maintain blood glucose stability by increasing insulin secretion, but when the compensatory capacity is exceeded, insulin resistance gradually worsens and eventually progresses to T2DM.

A study (51) collected more than 100,000 subjects with healthy blood glucose levels found that elevated TyG-BMI index increased the risk of early diabetes and that the overall risk was higher in women, people with normal, and persons aged below 50 years of age; in a retrospective cohort study in the same year (52), it was further suggested that the optimal TyG-BMI index for predicting an onset of diabetes was 213.2966 cutoff value. The reason for the large difference in TyG-BMI index and T2DM incidence by gender consider body composition and metabolic rate varies across gender and age groups. At baseline, individuals with normal blood glucose levels were enrolled and 8,430 males and 7,034 females were analyzed in another study based on a Japanese cohort (53); confirming a positive correlation between TyG-BMI index and the risk of T2DM in Japanese with healthy blood glucose levels, with an optimal threshold of 197.2987 and the risk was higher persons aged 18–44 years, females and non-hypertensive populations, and non-drinkers were more higher. In our study of a Chinese elderly population, it was revealed that TyG-BMI index was positively associated with T2DM incidence especially among the men and general subjects older than or equal to 75 years for reliable marker of early recognition of elderly at risk of T2DM. TyG-BMI index was proposed by Li et al. (54) as a valid marker for predicting diabetes in the impaired fasting glucose group (FBG 100–125 mg/dl or HbA 1c 5.7%–6.4%), but with higher predictive value in the normoglycaemic level group. A study in older US patients with DM found a U-shaped relationship between TyG-BMI index and all-cause mortality and a linear association with cardiovascular disease mortality (55). This shows that TyG-BMI index has a predictive value for both the occurrence of DM and adverse DM outcomes. Early management of this indicator may have a significant impact on the prevention of diabetes and complications.

## 7 TyG-BMI index and hyperuricemia

An association between human BMI and serum uric acid levels has been demonstrated (56). Obesity and overweight are frequently regarded as risk factors for hyperuricemia (HUA). Elevated uric acid not only contributes to gout and chronic kidney disease, but has also been shown to be an independent risk factor for cardiovascular disease. The IR and TyG-BMI indexes concerned in this paper are also inextricably linked to HUA. The pathological mechanism may be that insulin can affect the role of some urate transporters. When IR occurs in the body, insulin impairs the kidneys' ability to excrete uric acid, leading to uric acid accumulation in the body. Additionally, it damages the secretory function of pancreatic islet β cells, disrupts glucose and lipid metabolism, increases fatty acid production, inhibits insulin-mediated lipolysis and glucose uptake, causes purine metabolism disorders, and ultimately leads to elevated uric acid levels and the development of HUA.

The TyG index, TyG-BMI index, and TyG-WC index are useful for risk stratification and HUA prevention, according to Gu et al.'s study of 42,387 adults who had a routine physical examination and did not have HUA. The study also revealed that the TyG-BMI index was more detrimental for HUA in females than in males in the diseased group compared to the normal group, with a higher mean difference in females (51.90 for females and 36.90 for males) (57). Hao et al. (58) recruited 7,743 people (3,806 males, 3,937 females, mean age: 45.17 ± 17.10 years), of whom 32.18% had HUA. They studied the association between HUA risk and the TyG index, TyG-BMI index, TG/HDL-C ratio, and metabolic score of insulin resistance (METS-IR) in US patients without diabetes. The relationship between the TyG index, TyG-BMI index, TG/HDL-C and METS-IR was finally concluded that the risk of HUA was positively correlated with the elevation of TyG index, TyG-BMI index, TG/HDL-C and METS-IR in a large-scale population in the US. Further ROC curve analyses showed that TyG-BMI index and METS-IR were more capable of discriminating IR in both sex groups compared to TyG and TG/HDL-C, and that the combination of the obesity index with TyG index had better results; the ORs of the highest quartile of these two indexes for HU were TyG-BMI index: 7.15; METS-IR: 7.84; and this study also suggests a better predictive effect for women than men, considering the possibility that women are more sensitive to insulin due to different sex hormones and adipokines. Another study by Li et al. (59) investigated the predictive ability of TyG-BMI index in HUA combined with HTN similar to the above; TyG index, TyG-BMI index, TG/HDL-C, and METS-IR had significant correlation with hypertension combined with hyperuricemia, and TyG-BMI index and METS-IR had the ability to discriminate hypertension combined with hyperuricemia. Previous studies have consistently shown that patients with hypertension with hyperuricemia (HTN-HUA) have a higher risk of CVD than hypertensive patients with normal serum uric acid levels (60). A study on the ability of TyG-BMI index to predict HUA in middle-aged and elderly people (>45 years old) concluded that the risk of hyperuricemia in participants with the highest quartile of TyG-BMI index was 10.17 times that of the lowest quartile.

## 8 TyG-BMI index and fatty liver disease

Fatty liver disease (FLD) is a pathological condition of the liver characterized by excessive fat accumulation in liver cells. Its incidence is increasing and tends to occur at a younger age. Non-alcoholic fatty liver disease (NAFLD) can lead to hepatocellular carcinoma, and its common risk factors include insulin resistance, hyperlipidemia, and visceral obesity (61). The liver has a limited capacity to store triglycerides. During overeating, lipid deposition accelerates the rate of fatty acid β—oxidation, resulting in increased release of mitochondrial reactive oxygen species, aggravated oxidative damage. At the same time, it activates Kupffer cells and pro—inflammatory pathways and recruits immune cells, ultimately leading to liver cell damage (62). Numerous studies have confirmed that TyG-BMI index, a surrogate marker of IR, is strongly associated with NAFLD. Li et al. (63) through a secondary analysis of a prospective cohort study, concluded that the risk ratio of NAFLD increased with each SD increase in TyG-BMI index in a lipid-neutral and non-obese Chinese population, and the risk was higher in the female population (HR: 3.58, 95% CI: 2.80–4.60).

Nonetheless, it has been observed that individuals with non-obesity related NAFLD stand a high risk of developing metabolic disorders (64); Zhang et al. (65) employed ultrasound to identify a correlation between an increase in the incidence of NAFLD and a rise in the TyG-BMI index in participants who were not obese. Wang et al. (66) also discovered a well-defined positive correlation between NAFLD and the TyG-BMI index, as well as a stable nonlinear relationship with clear threshold and saturation effects. A threshold effect appeared when the TyG-BMI index was between 100 and 150, and the corresponding risk of NAFLD was saturated when it was between 300 and 400, the corresponding risk of NAFLD was saturated; AUC analysis showed that the TyG-BMI index was more predictive of NAFLD risk than other traditional indicators (*P* < 0.01), especially in young and middle-aged and non-obese populations. Using a sizable sample, Hu et al. (67) investigated the application of the TyG-BMI score to reliably distinguish between individuals with and without NAFLD, minimizing the need for extra screening in patients with low-risk NAFLD and minimizing needless ultrasound exams. Otsubo et al. (68) found in a retrospective observational study that the TyG-BMI index predicted NAFLD with a AUC values were significantly higher (AUC: 0.886, 95% CI: 0.8797–0.8927, *P* < 0.0001). These studies screened for fatty liver disease by simple biochemical indicators and took appropriate interventions to reduce fat accumulation in the liver and prevent further liver damage. The subgroup analysis of the predictive value of TyG-related parameters for fatty liver disease by Chen et al. showed that TyG-BMI index was more suitable for predicting all-cause mortality in patients without advanced fibrosis (69).

## 9 TyG-BMI index and metabolic bone disease

The mechanism of metabolic bone disease involves abnormalities in 3 main areas: bone resorption, bone development, and mineral deposition. Osteoporosis is a bone disease characterized by skeletal failure and osteoclast degeneration, leading to increased fracture incidence, cardiovascular disease, and mortality, and the association with TyG-BMI index cannot be ignored.

According to a recent study, in middle-aged and older Chinese people without diabetes, the TyG-BMI index was inversely correlated with fracture risk and positively correlated with bone mineral density (BMD) and geometric morphologic alterations (70). The findings of the few research that have been done on the connection between BMD and the TyG-BMI index are debatable. It has previously indicated that the TyG-BMI index is a trustworthy stand-in for IR. IR and BMD were directly correlated in a research by Francisco et al. (71) in postmenopausal women without diabetes, but there was no correlation between IR and osteoporosis prevalence. While Shin et al. (72) reported an inverse relationship between HOMA-IR and aBMD in a study of young Korean men (mean age 49.9 years), suggesting that IR is a negative predictor of bone health. Whether and how IR affects bone is controversial. The following has been taken into account in analyses: First, a high TyG-BMI index is typically linked to higher body fat, and by inducing chronic low-level inflammation, adipokine secretion, and estrogen synthesis, large amounts of adipose tissue may indirectly regulate bone metabolism (18); second, higher TyG-BMI index increases the levels of inflammatory factors such as IL-6 and TNF-α, and end products of glycosylation (18, 73), which triggers apoptosis and Wu et al. (74) suggested that there is a negative correlation between TyG-BMI index and testosterone in men, and normal testosterone levels are essential for male physiological processes, including sexual function, cardiovascular health, metabolism, brain function, and bone mineral density (75), therefore, an increase in TyG-BMI index and a decrease in testosterone levels in men may indirectly lead to a decrease in bone mineral density. can indirectly lead to decreased bone density and increased risk of osteoporosis in men.

## 10 TyG-BMI index and cancer

The TyG-BMI score is one of the common indicators of IR, and other earlier research have demonstrated a substantial correlation between IR and chances of developing cancer. As a result, numerous studies are investigating the association between cancer and the TyG-BMI index. Of these, few studies have verified the association between the TyG-BMI index and non-small cell lung cancer (NSCLC). Although there was no discernible variation in the TyG-BMI index levels at comprehensive TNM staging, case-control research by Wang et al. revealed that the index is a helpful tool for determining the risk of NSCLC (76). Using TyG-BMI index further to predict prognosis in advanced NSCLC, Guo et al. showed that the high TyG-BMI index group had a shorter overall survival period (77). And TyG-BMI index also has a correlation with oesophageal cancer (78). Additionally, the TyG-BMI index has prognostic value for pancreatic cancer paired with diabetes (79). Higher TyG-BMI index promotes more remodelling of the metabolic phenotype of pancreatic cancer cells increasing their aggressiveness (80). Given that insulin has the ability to cause cancer, hyperinsulinemia may increase the bioactivity of insulin-like growth factor I (IGFI), improve growth factor-dependent cell proliferation, and/or have a direct impact on cellular metabolism. The overproduction of ROS, which can harm DNA, cause mutations, and result in cancer, may increase the risk of cancer in patients with IR (81).

## 11 Summary and outlook

In summary, the TyG-BMI index, composed of three simple indicators:TG, FBG, and BMI, constructs a low-cost and easily accessible evaluation model by integrating routine biochemical indicators and weight data. In early screening, its sensitivity is significantly higher than that of single TG or FBG measurements, enabling the identification of high-risk populations during the asymptomatic phase of disease. Additionally, at the risk assessment level, evidence from multiple studies confirms that the risk of cardiovascular events, T2DM, NAFLD, and other conditions increases linearly with elevations in the TyG-BMI index. This quantitative association provides an accurate basis for clinical hierarchical management; in the formulation of prevention and treatment strategies, this index can dynamically monitor the intervention effect. For example, a decrease in TyG-BMI following weight loss, dietary control, or drug treatment indicates improved insulin sensitivity, with this change assisting doctors in timely adjusting the treatment plan. It is worth noting that height, weight, fasting blood glucose, and triglycerides are all routine screening indicators, and the data are easily accessible and cost-effective. This enables the TyG-BMI index to be seamlessly integrated into settings such as community health screenings and inpatient evaluations. Being particularly suitable for early detection of metabolic diseases in primary care institutions, it facilitates precise management of individual patients.

However, limitations exist in current research. Although numerous studies have confirmed the association between TyG-BMI and diseases, unified thresholds for different populations (e.g., children, the elderly, and special occupational groups) remain undefined, and its predictive value in rare or multi-system diseases requires further validation. In the future, there is an urgent need to conduct large-scale, multi-center, long-term follow-up epidemiological studies combined with genetic polymorphism analysis and metabolomics data, to clarify its biological mechanisms and develop personalized disease prevention and treatment strategies based on TyG-BMI, thereby promoting its translation from a research biomarker to a core clinical tool.
